# Development and validation of an interpretable prediction model for high-grade vesicoureteral reflux in children with urinary tract infections

**DOI:** 10.3389/fmed.2026.1864622

**Published:** 2026-10-02

**Authors:** Le-zhen Ye, Jian-xin Sun, Jing Chen, Jin-liang Chen, Ye Bi, Yan-ru Zhang

**Affiliations:** Department of Paediatrician, Women's and Children's Hospital of Ningbo University, Ningbo, China

**Keywords:** children, machine learning, SHAP, VCUG, vesicoureteral reflux

## Abstract

**Objective:**

High-grade vesicoureteral reflux (VUR) is prone to causing adverse clinical outcomes such as kidney damage, recurrent urinary tract infections, and hypertension. Early identification of the risk of developing high-grade VUR in children with urinary tract infections is of great significance for reducing unnecessary voiding cystourethrography (VCUG), guiding clinical decisions, and optimizing antibiotic management strategies.

**Methods:**

A total of 434 children with urinary tract infections who completed VCUG examination were retrospectively enrolled in this study. They were randomly divided into a training set (*n* = 304) and a test set (*n* = 130) in a 7:3 ratio. LASSO regression was used for feature selection. Based on the selected features, six machine learning prediction models were constructed: Logistic Regression, Random Forest, XGBoost, Gradient Boosting Machine, Elastic Net, and Support Vector Machine. The performance of the models was comprehensively evaluated using the area under the curve (AUC), integrated calibration index (ICI), precision-recall curve (PR), and decision curve analysis (DCA). Feature importance was ranked and explained using SHAP values.

**Results:**

Among the 434 patients, 156 individuals (35.9%) were diagnosed with grade ≥ 3 VUR. Seven predictive features were identified: delayed treatment response, annual UTI episodes, hydronephrosis, abnormal renal signal on MRU, febrile UTI (fUTI)/UTI ratio, age at first UTI, and non-Escherichia coli UTI episodes. Discrimination performance was comparable across the six models (test AUC range: 0.810–0.832; all pairwise DeLong tests *p* > 0.05); XGBoost was selected as the final model based on its good calibration (ICI=0.074) and consistent clinical net benefit, achieving a test AUC of 0.832 (95% CI: 0.757–0.897). Decision curve analysis showed that the model provided net benefits across threshold probabilities ranging from 0.15 to 0.90.

**Conclusions:**

We developed and validated a prediction model based on XGBoost, which can be used to assess the risk of high-grade vesicoureteral reflux in children with urinary tract infections. This model has good discrimination, acceptable calibration, and considerable clinical application potential. Additionally, based on this model, we developed and deployed a free online calculator at https://vur-prediction.yezhiqiu.cn, which can assist in real-time individualized risk assessment.

## Introduction

Vesicoureteral reflux (VUR) is one of the most common congenital structural abnormalities of the urinary system, with a prevalence of approximately 1% in the general pediatric population (1). However, in children with a history of febrile urinary tract infections (fUTI), this rate can reach 30% to 40% (2). The clinical consequences of VUR are closely related to its severity, with patients with higher-grade VUR having a significantly higher risk of developing renal scars and kidney damage compared to those with lower grades (3). Higher-grade VUR can progress to chronic kidney disease and is one of the main causes of pediatric end-stage renal disease (ESRD) (4). Therefore, the main goals of clinical management of VUR include early identification of high-risk children, prevention of recurrent urinary tract infections, and reduction of long-term kidney damage.

Studies have confirmed that age at first urinary tract infection ≤ 1 year, hydronephrosis, duplex collecting system, recurrent urinary tract infections, non-Escherichia coli infections, and renal ultrasound abnormalities are all independent predictors of VUR or high-grade VUR (5–7). However, the predictive value of each risk factor is limited, and no single variable can fully guide imaging decisions. Objective and quantitative risk stratification tools are crucial (8). VCUG is the gold standard for diagnosing VUR, but due to its invasiveness and radiation exposure, current guidelines only recommend this examination in specific circumstances, such as abnormal renal ultrasound, atypical pathogenic bacteria-induced infections, or complex clinical processes (9, 10). Although this method helps avoid unnecessary examinations, it may still miss some severely affected children with VUR who do not meet these criteria. A more comprehensive risk stratification tool may be able to fill this gap in clinical practice.

In recent years, machine learning has provided new strategies for addressing this clinical challenge (11). Compared with traditional logistic regression, machine learning algorithms can automatically capture the nonlinear relationships among multiple variables, demonstrating great potential (12, 13). Numerous studies have explored the application of machine learning in stratifying the risk of vesicoureteral reflux. However, most of the existing studies are limited by the design of a single algorithm and the lack of interpretability analysis, which hinders their widespread application in clinical practice (14).

Therefore, we retrospectively included 434 pediatric patients with urinary tract infections who underwent VCUG examinations. We used LASSO regression for feature selection and evaluated the predictive performance of six machine learning models: Logistic Regression (LR), Random Forest (RF), Extreme Gradient Boosting (XGBoost), Gradient Boosting Machine (GBM), Elastic Net (EN), and Support Vector Machine (SVM). Additionally, we applied SHAP analysis to interpret each feature's contribution in the optimal model and developed a free online calculator for clinical use.

## Materials and methods

### Study population

This study was approved by the Medical Ethics Committee of the Women's and Children's Hospital of Ningbo University (EC2023-011).

This study initially screened 550 pediatric patients who underwent VCUG examinations at the Women's and Children's Hospital of Ningbo University from January 2019 to October 2025. Patients were eligible for inclusion if they: (1) were aged ≤ 18 years; and (2) had at least one confirmed episode of urinary tract infection prior to VCUG examination.

The following exclusion criteria were applied: (1) VCUG performed for indications other than UTI-related VUR risk evaluation, including follow-up of previously diagnosed VUR, antenatal hydronephrosis, or other structural urinary tract anomalies (*n* = 89);(2) History of prior urinary tract surgery (*n* = 26);(3) Missing VCUG imaging data (*n* = 1).

Following the application of these exclusion criteria, 116 patients were excluded, resulting in a final analytical cohort of 434 patients. The patient selection process is illustrated in Figure 1.

**Figure 1:**
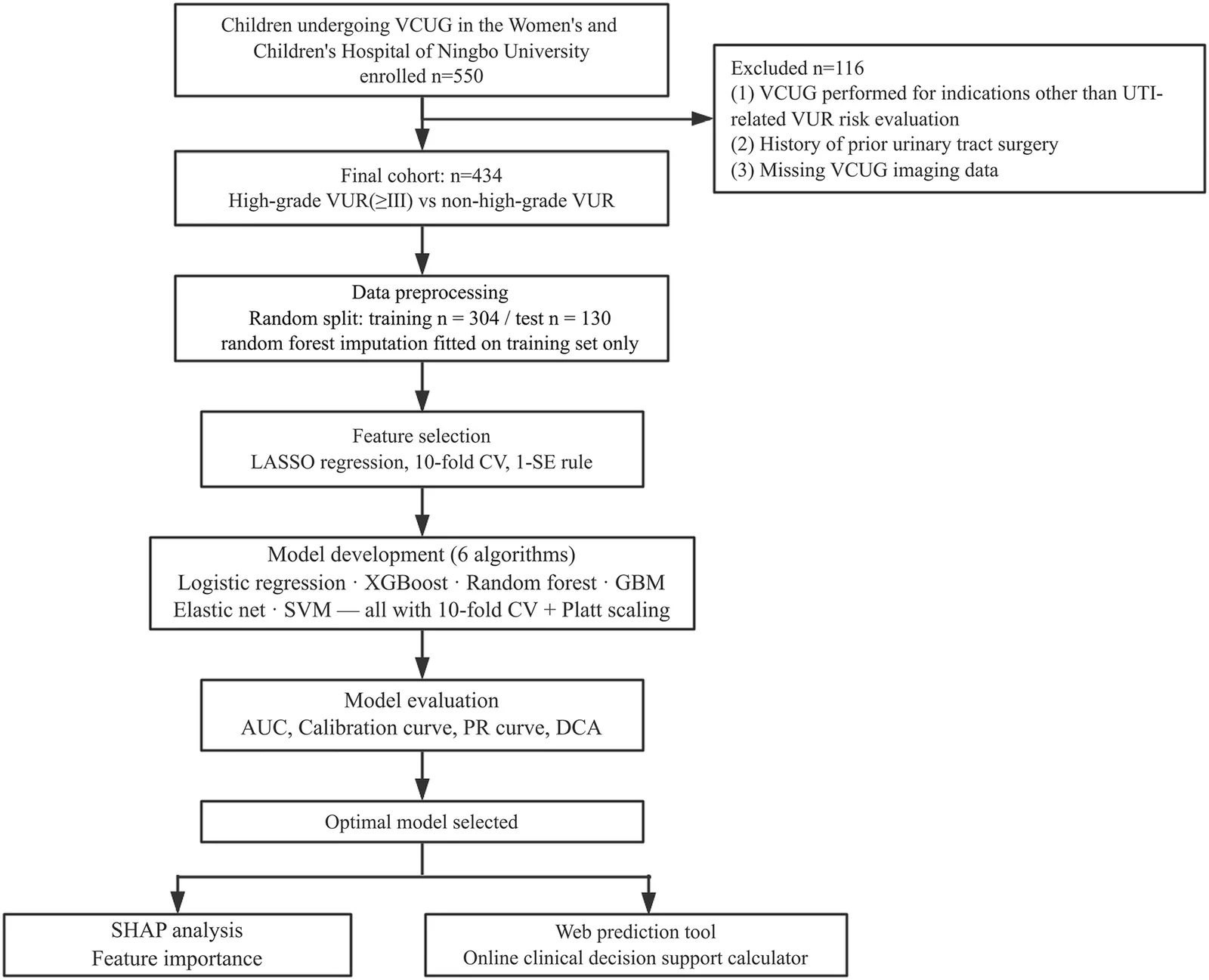
Flowchart of patient selection and study workflow.

### Definition of outcome variables

All pediatric patients were classified into high-grade reflux and non-high-grade reflux groups based on VCUG results. VUR was graded according to the International Reflux Study Committee classification system, which comprises five grades: Grade I, reflux into a non-dilated ureter; Grade II, reflux into the renal pelvis and calyces without dilatation; Grade III, mild-to-moderate dilatation of the ureter and pelvicalyceal system; Grade IV, dilatation of the renal pelvis and calyces with moderate ureteral tortuosity and blunting of the fornices; and Grade V, gross dilatation of the ureter, pelvis, and calyces with loss of papillary impressions and severe ureteral tortuosity (11). High-grade VUR was defined as reflux grade ≥ 3.

### Study indicators

The following 13 variables were extracted and analyzed: age at first urinary tract infection (UTI), gender, bladder and bowel dysfunction, estimated glomerular filtration rate (eGFR), body weight status, annual incidence of UTIs, proportion of fUTIs among total UTIs, delayed response to treatment, number of non-Escherichia coli UTI episodes, fetal urinary tract ultrasound abnormalities detected during prenatal checkups, hydronephrosis, abnormal renal signals on MRU and observation window (the interval between the first UTI and VCUG). We included all the above variables in the initial feature set and performed variable selection using LASSO regression. Variables were defined as follows:

Age at first UTI: Age (in months) at the time of the first confirmed urinary tract infection. Diagnosis of UTI was made according to the 2021 ESPU Guidelines (15).
Gender: Recorded as a binary classification (male or female).
Bladder and bowel dysfunction (BBD): Defined as positive if medical records documented both urinary symptoms (frequency, urgency, incontinence, delayed voiding, etc.) and bowel dysfunction (constipation, fecal incontinence, etc.).
Estimated glomerular filtration rate (eGFR): Calculated using the revised Schwartz formula: eGFR = k × 88.4 × height (cm)/serum creatinine (*μ*mol/L). Serum creatinine was measured by the Jaffe method. The coefficient k was set at 0.45 for infants aged 1–12 months, 0.55 for children aged 2–12 years, 0.77 for males aged >12 years, and 0.55 for females aged >12 years. Results were reported in mL/min/1.73 m^2^. An eGFR <90 mL/min/1.73 m^2^ was considered abnormal.
Weight status: Assessed using weight-for-age Z-scores (WAZ) based on the 2006 WHO child growth standards. Subjects were classified into three categories: overweight (WAZ > + 1 SD), normal weight (−1 SD ≤ WAZ ≤  + 1 SD), and underweight (WAZ < −1 SD).
Annual incidence of urinary tract infection: Defined as the total number of confirmed urinary tract infections per year. For children with first-time UTI before 1 year of age, the annual incidence was calculated by extrapolating from the number of infections during the available observation period (annual incidence=actual number of infections ÷ observed months × 12).
fUTI/UTI ratio: The ratio of the number of fUTI episodes to the total number of UTIs.
Given that observation windows before VCUG varied across patients, we examined the association between window length and UTI-related features, included the window length itself as a candidate variable, and conducted a sensitivity analysis excluding patients with short observation windows (<3 months) to confirm the robustness of model performance.
Delayed response to treatment: Defined as failure of body temperature to normalize (≥ 37.5 °C) within 48 h after initiation of empirical antimicrobial therapy.
Number of non-Escherichia coli UTI episodes: The cumulative number of episodes where previous urine culture results indicated infection by non-Escherichia coli pathogens.
Fetal urinary tract ultrasound abnormalities detected: Based on the mother's prenatal records, the presence of renal pelvic dilatation (anterior-posterior diameter ≥ 4 mm) or other urinary system structural abnormalities detected by prenatal ultrasound was considered a positive criterion.
Hydronephrosis status: Based on ultrasound and MRU findings, hydronephrosis was graded according to the Urinary Tract Dilatation (UTD) classification system (16); a positive result was defined as the presence of hydronephrosis detected by either ultrasound or MRU. In this study, 45 children (10.4%) lacked MRU data; for these cases, the ultrasound results were used to determine the hydronephrosis status. Based on an analysis of 389 children with complete data from both ultrasound and MRU, the false-negative rate of ultrasound for hydronephrosis detected by MRU was 9.4% (28/299), suggesting that hydronephrosis may be underestimated in a small number of children with negative ultrasound findings and missing MRU data.
Abnormal renal signal on MRU: Abnormal renal parenchymal signal on magnetic resonance imaging was used as the criterion for a positive result, reflecting renal parenchymal damage. MRU was performed using a contrast-free, water-based (heavily T2-weighted) imaging protocol, without intravenous gadolinium administration. In this study, MRU data were missing for 45 cases and were imputed using the random forest method (see Section 1.4 for details). At our institution, MRU was not obtained as a routine baseline investigation in all children with UTI, but was selectively performed in those with recurrent urinary tract infections, febrile UTI, or abnormal findings on renal ultrasound; this selective use should be considered when generalizing this predictor to settings with different MRU indications.

VCUG and MRU images were evaluated separately by two independent radiologists, with discrepancies adjudicated by a third senior radiologist. Ultrasound assessment of hydronephrosis was performed by a single radiologist as part of routine clinical workflow.

Inter-rater agreement for VUR grading between the two primary radiologists was assessed at the renal-unit level using linear-weighted Cohen's *κ*, with 95% confidence intervals obtained via patient-clustered bootstrap resampling (1,000 iterations) to account for within-patient correlation between bilateral renal units. Agreement was excellent across the full grading scale (*κ* = 0.976, 95% CI: 0.969–0.984, based on 868 renal units from 434 patients) and remained strong when restricted to reflux-positive renal units only (*κ* = 0.913, 95% CI: 0.881–0.941; 87.7% exact agreement).

### Construction and evaluation of predictive models

We constructed and validated machine learning models using R (version 4.4.1) and Python (version 3.11.9). A standardized data preprocessing workflow was applied, including dataset splitting, missing-data imputation, and feature selection. Detailed procedures are described below:

Data Partitioning: Using the createDataPartition function from the R software package caret (version 7.0.1), 434 patients were randomly divided into a training group (*n* = 304) and a test group (*n* = 130) in a 7:3 ratio.
Missing Value Handling: MRU signal was missing in 45 patients (10.4%). To keep the test set independent during preprocessing, missing values were imputed after the train/test partition: a random forest classifier (randomForest package, 500 trees) was fitted exclusively on the complete cases of the training set and then applied to both the training set (*n* = 32 imputed) and the test set (*n* = 13 imputed) without refitting. The imputation model included only the other twelve candidate predictor variables described in Section 1.3 and did not include the outcome variable (VUR grade), thereby avoiding outcome-related information leakage. To verify the stability of the imputation approach, we conducted a sensitivity analysis under two alternative assumptions, applied after the train/test partition: (i) all missing MRU values were set to normal, and (ii) missing values were randomly assigned according to the abnormal rate observed in the training set. LASSO selection and model training were repeated independently in each scenario. The test AUC differed by less than 0.02 across scenarios (Logistic regression, 0.810–0.813; XGBoost, 0.820–0.828), and MRU signal was retained by LASSO in all scenarios, confirming the reliability of the imputation strategy.
Feature Selection: LASSO regression (glmnet package, version 4.1.8) was applied to all candidate clinical variables in the training set. The optimal regularization parameter *λ* was determined through 10-fold cross-validation. We adopted the 1-Standard Error (1-SE) criterion, and variables with non-zero coefficient estimates were retained as the final features for building the model.
Model Construction and Calibration: Based on the selected features, six machine learning models (LR, RF, XGBoost, GBM, EN, and SVM) were constructed using the caret package in R (version 7.0.1). We performed hyperparameter tuning for all models through 10-fold cross-validation. To verify that model selection was not driven by test-set-specific information, we additionally compared all six algorithms using cross-validated AUC within the training set alone.
Some algorithms, especially those based on trees, often have problems with probability calibration bias. To address this issue, we employed Platt scaling to calibrate the predicted probabilities of the six models. We used the predicted results from the training set to fit a logistic calibration model, and then applied this model to adjust the predicted probabilities of the test set. In terms of performance evaluation, calibration performance was assessed using ICI, while clinical practicability was evaluated using DCA and the area under the precision-recall curve (AUPRC) — all of which were calculated using the calibrated probabilities. For discrimination performance, we used pre-calibrated probabilities to calculate AUC, enabling a direct comparison of the discrimination capabilities of each model.
Overall Model Performance Evaluation: The ROC curve and AUC value were calculated using the pROC package in R (version 1.18.5), and the 95% confidence interval was estimated through bootstrap sampling (1,000 iterations). The calibration curve was plotted using the lowess function to assess the calibration performance, and the ICI was used to quantify it. Additionally, we performed DCA with the ggplot2 package (version 3.5.2) across different threshold probabilities. The PRROC package (version 1.4) was used to calculate the precision-recall curve and AUPRC to evaluate the model performance under class imbalance. Finally, to assess the stability of the single train/test split, we performed 100 repeated random 70/30 stratified splits using the same features and hyperparameters, evaluating test AUC in each repetition.
SHAP Analysis: After determining the optimal model, we conducted a SHAP analysis using Python (version 3.11.9) and ranked features by their importance.

### Statistical analysis

Categorical variables were presented as frequencies and percentages. Comparisons were made between the high-grade vesicoureteral reflux group and the non-high-grade vesicoureteral reflux group, as well as between the training set and the test set, for these variables using the chi-square test or Fisher's exact test. Continuous variables were expressed as median and interquartile range, and pairwise comparisons were conducted using the Mann–Whitney U test. All tests were two-tailed, and a *P* value < 0.05 was considered statistically significant. Statistical analysis was performed using IBM SPSS Statistics (version 29.0.2).

## Results

### Baseline characteristics of the study population

This study included a total of 434 patients, with 304 in the training set and 130 in the test set. Although there was a statistically significant difference in the distribution of weight status between the two groups (*p* = 0.020), this variable was not selected as the final predictive feature by the LASSO regression algorithm and thus had no impact on model construction. Table 1 summarizes the baseline characteristics of the two groups. Beyond the distribution of weight status, there were no statistically significant differences between the training and testing sets for any other variables (all *p* > 0.05), which confirms that the two groups were comparable.

**Table 1:** Baseline clinical characteristics of patients in the training and test sets.

Variable	Training set (*n* = 304)	Test set (*n* = 130)	Statistic	*p*
Reflux group, *n* (%)				
Yes	105 (34.5)	51 (39.2)	0.870	0.351
No	199 (65.5)	79 (60.8)		
Gender, *n* (%)				
Male	134 (44.1)	60 (46.2)	0.159	0.690
Female	170 (55.9)	70 (53.8)		
Age at first urinary tract infection, *n* (%)				
< 6 months	131 (43.1)	63 (48.5)	1.540	0.463
6 months to 1 year	39 (12.8)	18 (13.8)		
> 1 year	134 (44.1)	49 (37.7)		
Weight status, *n* (%)				
WAZ > + 1SD	68 (22.4)	29 (22.3)	7.797	0.020
−1SD ≤ WAZ ≤ + 1SD	185 (60.9)	92 (70.8)		
WAZ < −1SD	51 (16.8)	9 (6.9)		
BBD, *n* (%)				
Present	19 (6.3)	4 (3.1)	1.827	0.177
Absent	285 (93.8)	126 (96.9)		
Abnormal fetal urinary system ultrasound findings, *n* (%)				
Abnormal	17 (5.6)	7 (5.4)	0.008	0.931
Normal	287 (94.4)	123 (94.6)		
Delayed response to treatment, *n* (%)				
Yes	66 (21.7)	30 (23.1)	0.099	0.753
No	238 (78.3)	100 (76.9)		
Hydronephrosis, *n* (%)				
Abnormal	83 (27.3)	43 (33.1)	1.474	0.225
Normal	221 (72.7)	87 (66.9)		
Abnormal eGFR, *n* (%)				
Yes	70 (23.0)	39 (30.0)	2.355	0.125
No	234 (77.0)	91 (70.0)		
Abnormal renal signal on MRU, *n* (%)				
Abnormal	105 (34.5)	41 (31.5)	0.367	0.544
Normal	199 (65.5)	89 (68.5)		
Non-E. coli UTIs, *n* (%)				
None	259 (85.2)	106 (81.5)	0.912	0.340
≥ 1 episode	45 (14.8)	24 (18.5)		
Proportion of fUTIs (fUTI/UTI), median (IQR)	0.500 (0.000, 1.000)	1.000 (0.000, 1.000)	−0.742	0.458
Annual UTI incidence (episodes/year), median (IQR)	1.500 (0.342, 4.000)	2.150 (0.372, 4.575)	−0.923	0.356
Observation window (months), median (IQR)	2.000 (0.000, 8.000)	2.000 (1.000, 8.000)	−0.187	0.852

Chi-square test was used for categorical variables; Fisher's exact test was applied when expected cell counts were fewer than 5, in which case no test statistic is reported; Mann–Whitney U test was used for continuous variables.

### LASSO regression results

Following 10-fold cross-validation in the LASSO regression analysis, the optimal regularization parameter *λ* was determined using the 1-SE criterion (*λ*.1se = 0.0425) (Figure 2A), resulting in the inclusion of 7 predictive factors: delayed response to treatment, annual UTI episodes, hydronephrosis, abnormal renal signal on MRU, fUTI/UTI ratio, age at first UTI, and non-E. coli UTI episodes (Figure 2B). The distribution of these 7 variables between the high-grade VUR and non-high-grade VUR groups is shown in Figure 2C.

**Figure 2:**
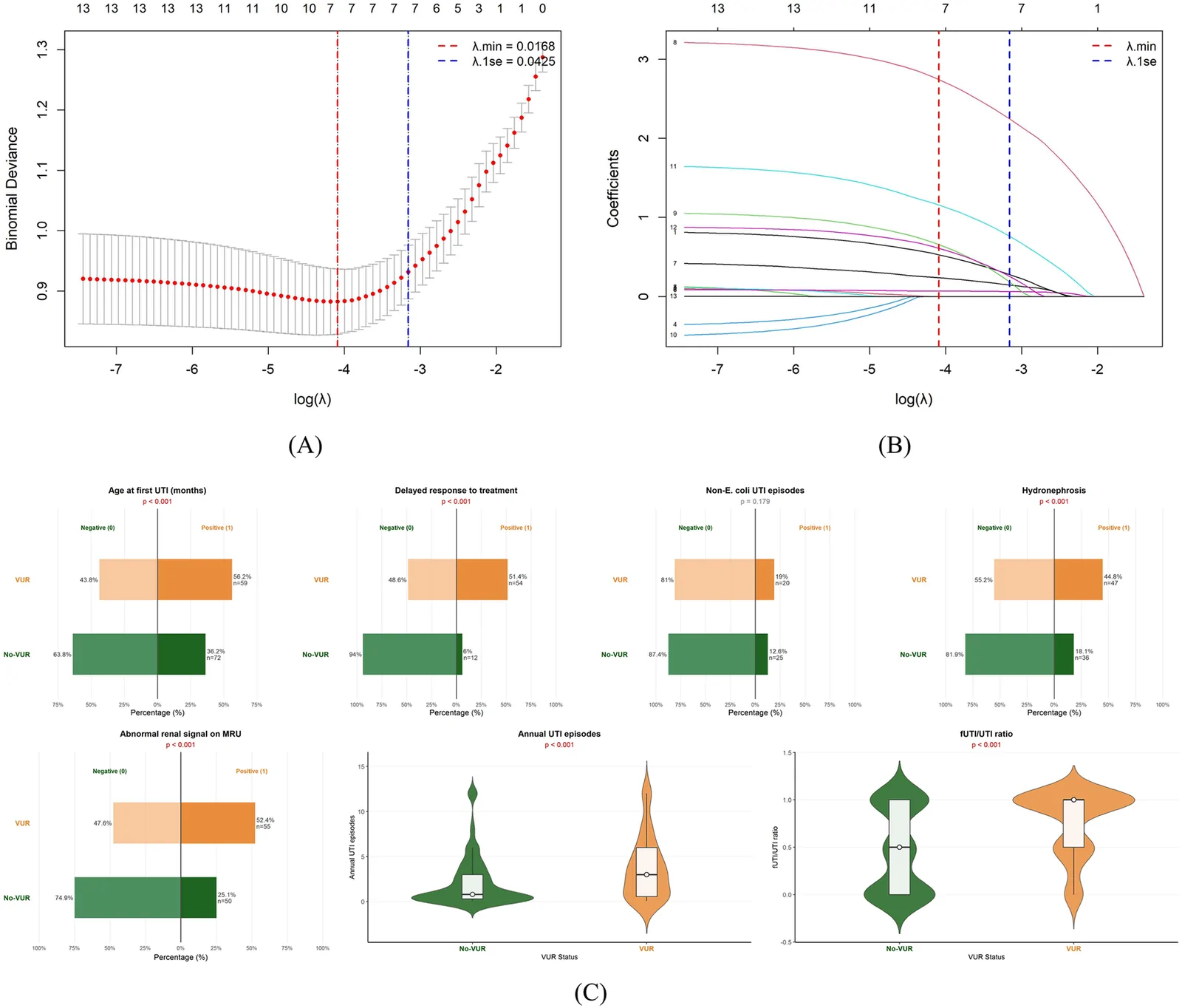
LASSO regression with 10-fold cross-validation. **(A)** Cross-validation deviance curve. The left dashed line indicates *λ*.min = 0.0168 (minimum cross-validation error); the right dashed line indicates *λ*.1se = 0.0425 (1-SE criterion), which was adopted in this study. **(B)** LASSO coefficient paths. Each colored line represents one variable; coefficients shrink toward zero as *λ* increases. The 7 non-zero coefficients retained at *λ*.1se correspond to the selected predictive features. **(C)** The distribution of 7 characteristics across the high-grade vesicoureteral reflux group and the non-high-grade vesicoureteral reflux group. Categorical variables are presented in the form of proportion bar charts, while continuous variables are displayed using violin plots to illustrate their distribution patterns.

Annual UTI incidence and the fUTI/UTI ratio were both correlated with observation window length; after excluding patients with observation windows <3 months, this correlation disappeared for UTI incidence but persisted for the fUTI/UTI ratio. A sensitivity analysis excluding these patients (224/434 patients removed) showed stable model performance (test AUC 0.853 vs. 0.832).

### Comparative performance of machine learning models

Discrimination performance was comparable across the six models on the test set (AUC range: 0.810 -0.832) (Figure 3A, B); pairwise DeLong tests confirmed that none of these differences reached statistical significance (all *p* > 0.05; Table 2). To assess whether this result was sensitive to the specific train/test partition, we performed 100 repeated random 70/30 stratified splits using the same features and hyperparameters; the mean test AUC across repeats was 0.855 (SD = 0.027), with the originally reported AUC (0.832) falling within the lower range of this distribution, confirming that our result was not a favorably biased single-split estimate (Figure 3C). Given this comparable discrimination, model selection was guided primarily by calibration and clinical utility: XGBoost showed good calibration performance (ICI = 0.074, the lowest among the tree-based ensembles) (Figure 3D) and maintained consistent net benefit in DCA within the probability range of 0.15 -0.90 (Figure 3E). Although the AUPRC of RF was slightly higher than that of XGBoost (0.800 vs. 0.796) (Figure 3F), XGBoost's better calibration (ICI 0.074 vs. 0.099) was considered the decisive factor, and it was therefore selected as the optimal model.

**Figure 3:**
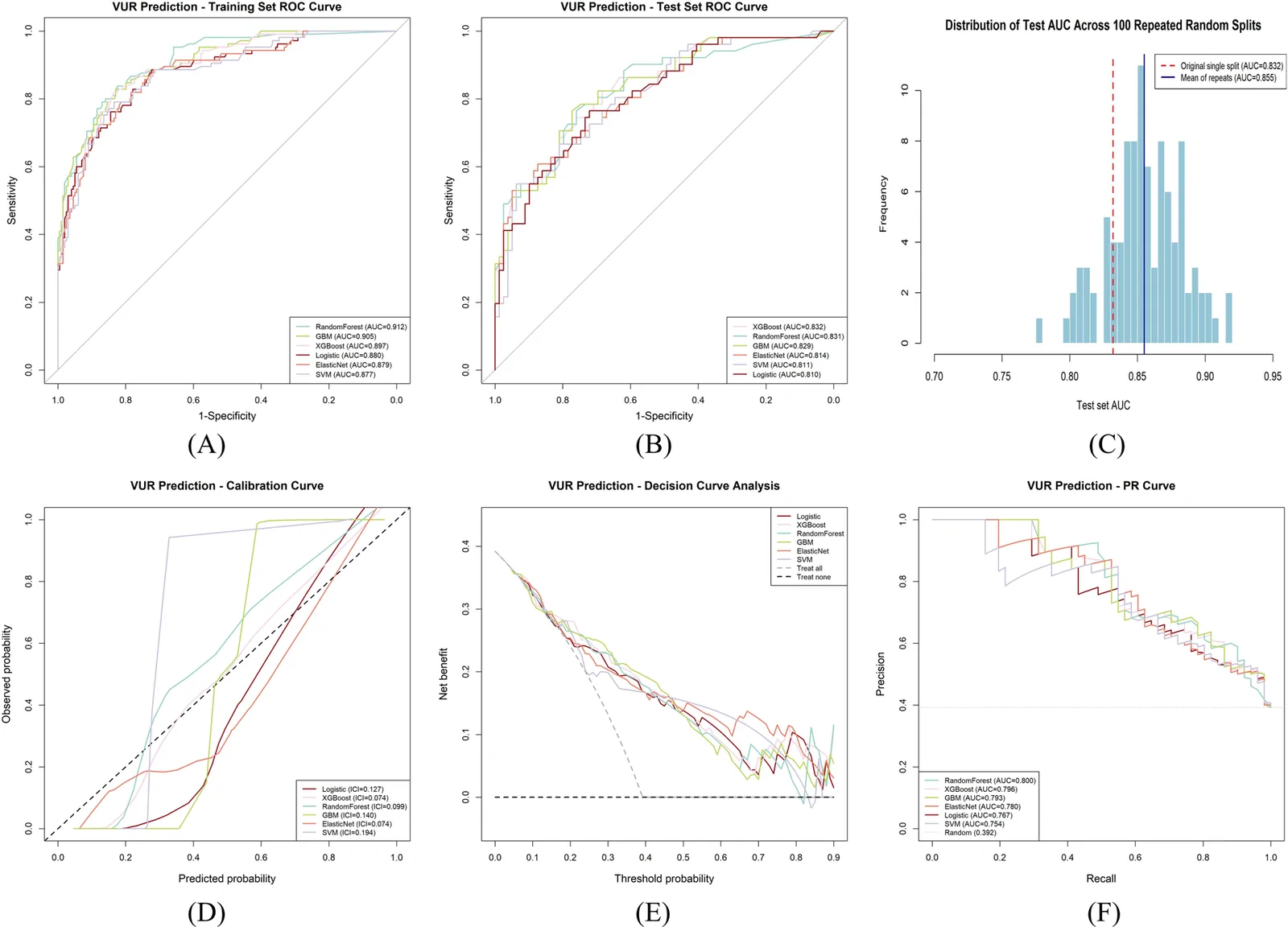
Presents the performance evaluation of six machine learning models for predicting high-grade VUR in children with UTI. **(A)** Training set ROC curves, showing the discriminatory power of each model, with GBM and RF achieving the highest AUCs. **(B)** Test set ROC curves(apparent performance from the final refitted models), with XGBoost achieving the numerically highest AUC (0.832), though discrimination was statistically comparable across models (Table 2). **(C)** Distribution of test AUC across 100 repeated random 70/30 splits, confirming that the originally reported AUC (0.832) was not a favorably biased estimate (mean = 0.855, SD = 0.027). **(D)** Calibration curves, with XGBoost showing good calibration (ICI = 0.074). **(E)** Decision curve analysis (DCA), indicating XGBoost and GBM had the most robust net clinical benefit within a relevant threshold range. **(F)** Precision-Recall Curves, with RF and XGBoost achieving the highest AUPRCs, and all models outperforming the random baseline. Comprehensive performance metrics for all models are summarized in Table 2.

**Table 2:** Performance comparison of different machine learning prediction models.

Model	Training CV-AUC (SD)^a^	Apparent AUC^b^	Test AUC (95% CI)	Test Sens.	Test Spec.	ICI	AUPRC	DeLong *p*^c^
XGBoost	0.878 (0.058)	0.897	0.832 (0.757–0.897)	0.824	0.684	0.074	0.796	-
RF	0.863 (0.059)	0.912	0.831 (0.757–0.898)	0.765	0.759	0.099	0.800	0.941
GBM	0.879 (0.050)	0.905	0.829 (0.750–0.891)	0.765	0.772	0.140	0.793	0.665
EN	0.869 (0.059)	0.879	0.814 (0.735–0.889)	0.549	0.937	0.074	0.780	0.139
SVM	0.868 (0.060)	0.877	0.811 (0.735–0.882)	0.549	0.937	0.194	0.754	0.144
LR	0.865 (0.090)	0.880	0.810 (0.730–0.884)	0.765	0.722	0.127	0.767	0.092

a Training CV-AUC: mean 10-fold cross-validated AUC obtained during hyperparameter tuning within the training set alone; logistic regression was cross-validated manually using identical fold partitions for consistency.

b Apparent AUC: AUC of the final refitted model evaluated on the same training data used to fit it (resubstitution performance); reported for reference only and not used for model selection.

c *P* values from pairwise DeLong tests comparing each model's test AUC to that of XGBoost; none reached statistical significance.

### SHAP analysis

Using the seven features determined by LASSO regression, we conducted SHAP analysis on the XGBoost model. The results showed the importance ranking of each feature as follows (Figure 4A): delayed response to treatment, annual UTI episodes, hydronephrosis, abnormal renal signal on MRU, fUTI/UTI ratio, age at first UTI, and non-E. coli UTI episodes. The swarm plot indicated that higher values of delayed response to treatment, annual UTI episodes, hydronephrosis, and abnormal renal signal on MRU were associated with higher SHAP values, suggesting that these features contributed positively to the model's prediction of high-grade VUR (Figure 4B). The fUTI/UTI ratio was also positively correlated with the predicted risk, but this association was less stable. It is worth noting that a younger age at the first urinary tract infection (lower feature value) was associated with a higher predicted risk, which is consistent with clinical findings – namely, that early occurrence of urinary tract infections is a risk factor for high-grade VUR. The SHAP heatmap across individual test-set instances confirmed that delayed response to treatment and annual UTI episodes contributed the largest and most consistent impact on the model output, in agreement with the feature importance ranking (Figure 4C).

**Figure 4:**
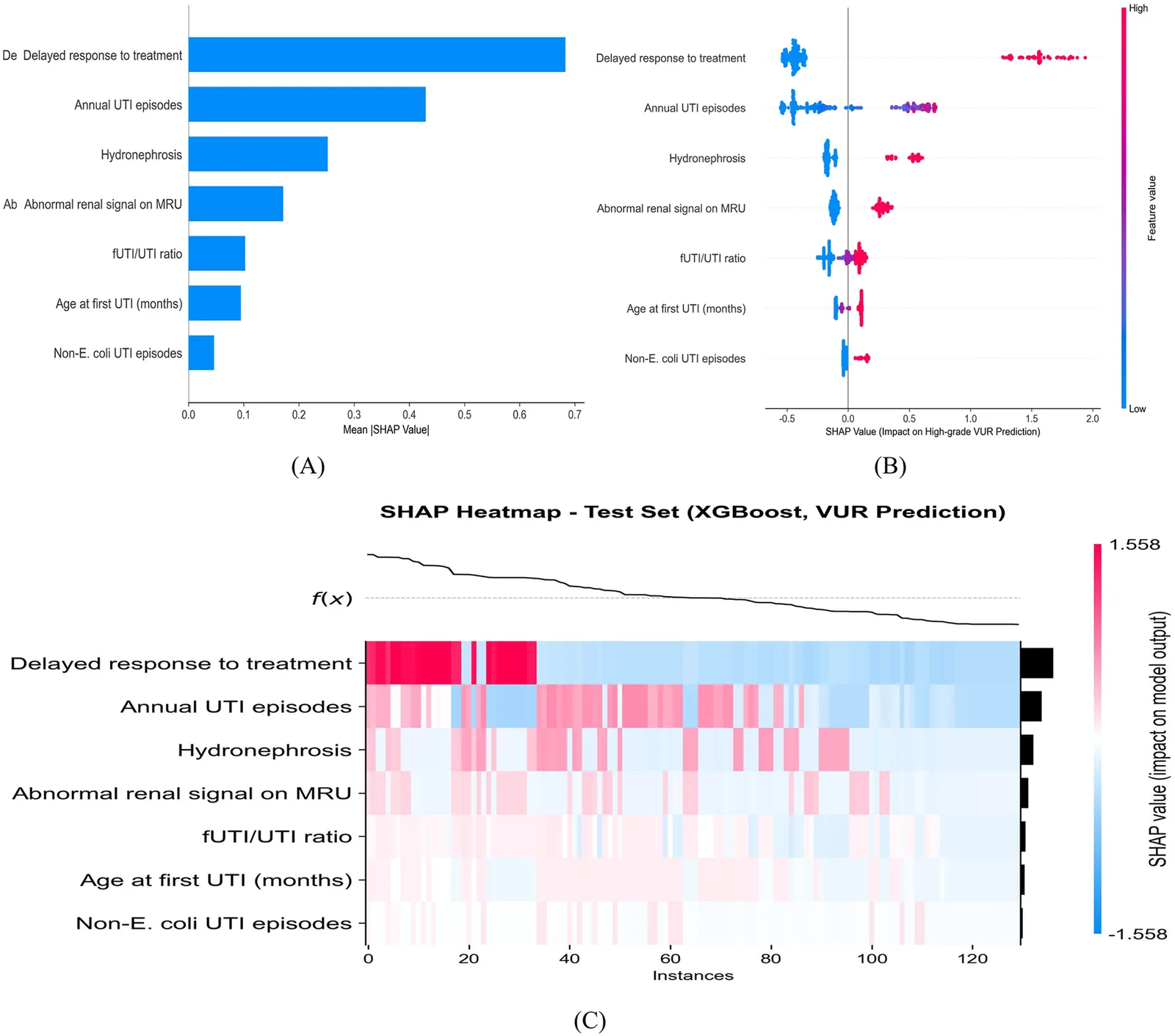
Presents the interpretation results of the XGBoost model based on SHAP. **(A)** Bar chart of the average absolute SHAP values, sorted by importance for the seven features. **(B)** Swarm plot, showing the direction and degree of influence of each feature on the prediction (high values are represented in red, and low values in blue). **(C)** SHAP heatmap for all test set instances (*n* = 130), sorted in descending order of the predicted probability of high-risk VUR, where the color intensity reflects the size of the SHAP contribution of each feature to the individual prediction.

### Web prediction tool

To support the application of our predictive model in clinical practice, we developed a user-friendly, web-based VUR risk calculator, accessible at https://vur-prediction.yezhiqiu.cn. This online tool integrates seven key clinical characteristics determined through LASSO regression analysis and the optimized XGBoost algorithm. Clinicians can obtain real-time and personalized probability estimates for severe VUR by entering relevant information for individual patients.

## Discussion

The current clinical guidelines recommend performing VCUG in specific situations, such as the first episode of acute pyelonephritis not caused by Escherichia coli, elevated plasma creatinine levels, or persistent moderate to severe hydronephrosis detected during postnatal ultrasound examinations—especially in patients with a family history of VUR (17, 18). However, as an invasive examination, VCUG involves radiation exposure and the risk of catheter-associated urinary tract infection (19), so it is necessary to accurately determine its indications. The predictive model developed in this study aims to provide clinicians with an objective and quantitative risk stratification tool. Its purpose is to identify high-risk children with severe VUR who truly need to undergo VCUG, thereby reducing unnecessary invasive examinations and lowering the risk of missed diagnoses.

In this study, the delayed therapeutic response emerged as the highest-scoring predictor, which is consistent with the results of a multicenter study involving 421 children with their first episode of acute pyelonephritis (20). Given that severe vesicoureteral reflux significantly increases the risk of renal scarring after acute pyelonephritis (21), the delayed therapeutic response indicated that the children needed to undergo timely imaging assessment.

The conclusion that recurrent urinary tract infections are an important risk factor for vesicoureteral reflux (VUR) has been confirmed by existing literature. A study involving 399 patients with primary VUR found that recurrent urinary tract infections are an important indicator for performing VCUG, and local renal scars are independently associated with recurrent urinary tract infections (OR = 1.8, *p* = 0.004) (22). This result provides evidence for the strong predictive weight of the annual incidence of urinary tract infection and the fUTI/UTI ratio in the SHAP analysis of this study. Prioritizing imaging evaluations for these children helps to reduce unnecessary examinations and simultaneously lowers the risk of missing advanced VUR.

Imaging examinations play a crucial role in the clinical assessment of VUR. Hydronephrosis has been regarded as a key indicator in the imaging assessment of VUR, and the incidence of urinary tract dilation is extremely high in patients with high-grade VUR (23). Although conventional ultrasound is widely used due to its ease of use and non-radiation nature, its sensitivity in diagnosing reflux remains limited (24, 25). Magnetic resonance urography (MRU) enables detailed assessment of abnormal renal parenchymal signals and hydronephrosis, and performs exceptionally well in detecting renal involvement and even scarring after urinary tract infections (26). Therefore, we included the abnormal renal signal patterns on MRU as a predictive indicator. Through LASSO regression analysis, this indicator was ultimately identified as a key predictive feature, a finding that has not been reported in existing prediction tools (27). Our study showed that MRU-derived abnormal signal patterns can serve as an independent radiological predictor for high-grade VUR, providing a valuable reference for institutions that cannot perform radionuclide scans.

The age at first UTI was also an important related factor. A retrospective study showed that age ≤1 year at first UTI was an independent risk factor for VUR in children, and the younger the age, the higher the detection rate of VUR (28)—which is consistent with the findings of our study.

The persistent association between shorter observation windows and higher fUTI/UTI ratios likely reflects referral patterns in clinical practice: children presenting with more severe, febrile infections may be referred for VCUG more promptly, while those observed over longer periods accumulate a higher proportion of milder infections. This pattern, rather than indicating measurement bias, may itself carry clinical relevance regarding referral urgency and disease severity.

Although non-E. coli infections did not reach the traditional statistical significance level (in the univariate analysis, *P* > 0.05), the LASSO regression algorithm still included them based on their overall contribution to the model's discriminative ability. Clinically, non-E. coli urinary tract infections are closely related to high-grade vesicoureteral reflux (VUR) (29), and the ESPU guidelines explicitly recommend performing VCUG in such cases (15). In contrast, another study showed that for the first episode of fUTI caused by E. coli, VCUG is not routinely necessary (30). This further emphasized the unique clinical significance of the pathogen type in predicting VUR.

The existing literature has clearly demonstrated a close association between BBD and UTI (31). According to the 2024 guidelines of the European Association of Urology and the European Society of Paediatric Urology, BBD is more common in children with VUR who have undergone toilet training, and the coexistence of BBD and VUR doubles the risk of urinary tract infection recurrence (32). Urodynamic assessment and effective treatment of BBD also contribute to the alleviation of VUR (33). However, our study did not identify BBD as a risk factor for high-grade VUR, which may be due to the younger age of our research subjects (in this age group, the symptoms of BBD have not yet fully manifested) and the lack of standardized urodynamic assessment in the retrospective records. Future studies should introduce standardized BBD scoring tools to address this limitation.

Beyond childhood renal scarring, severe VUR carries lasting adverse effects even after successful surgical correction. A 15-year follow-up cohort after Cohen ureteroneocystostomy found that nearly one-quarter of patients developed adult lower urinary tract dysfunction, with childhood bladder-bowel dysfunction, bilateral disease, renal scarring, female sex, and older age at surgery as independent risk factors; women who later became pregnant, particularly those with renal scarring or bilateral disease, remained at elevated risk of febrile UTI and pre-eclampsia (34). These findings highlight the importance of identifying occult high-grade VUR early, before its long-term consequences accumulate. The risk stratification model developed in this study offers a practical means of achieving this, allowing timely imaging and intervention in children who would otherwise be missed under current criteria.

## Limitations

Our study had several limitations. First, as a single-center retrospective study with a limited sample size, its external generalizability needs to be confirmed through future multicenter prospective validation. In addition, our cohort included only children who had already undergone VCUG, so the model reflects a preselected population rather than the full spectrum of children presenting with UTI. Whether it can reliably guide the initial decision to perform VCUG will require validation in a broader, unselected UTI population. Second, final selection between the two leading candidates (XGBoost and GBM) relied in part on their calibration and net-benefit performance in the test set; although both were confirmed as top performers using training-set cross-validation alone, this test-set-informed tie-breaking step may introduce a modest degree of optimism in the reported AUC. Third, the interpretation of certain predictive indicators (such as BBD) depended on the quality of medical record documentation, which may introduce information bias. Fourth, 45 patients (10.4%) had missing MRU data, so we relied solely on ultrasound for hydronephrosis assessment. Based on patients with complete data, the estimated false-negative rate of ultrasound relative to MRU was 9.4%, indicating that hydronephrosis may have been underestimated in some cases. Future studies should also incorporate standardized prospective data collection protocols and longer follow-up periods to enable causal inference and longitudinal outcome assessment.

## Data Availability

The raw data supporting the conclusions of this article will be made available by the authors, without undue reservation.
